# Supplementary material for: The causal role of circulating inflammatory markers in osteoporosis: a bidirectional Mendelian randomized study
Source: Front Immunol. 2024 Jul 18;15:1412298. doi: 10.3389/fimmu.2024.1412298 (PMC11291241; doi:10.3389/fimmu.2024.1412298)
Supplement: Supplementary file 1 [file DataSheet_1.zip › supplementary material/OSTEOPOROSIS-CIM/GCST90274782.tsv.gz_leave_one_out.pdf]

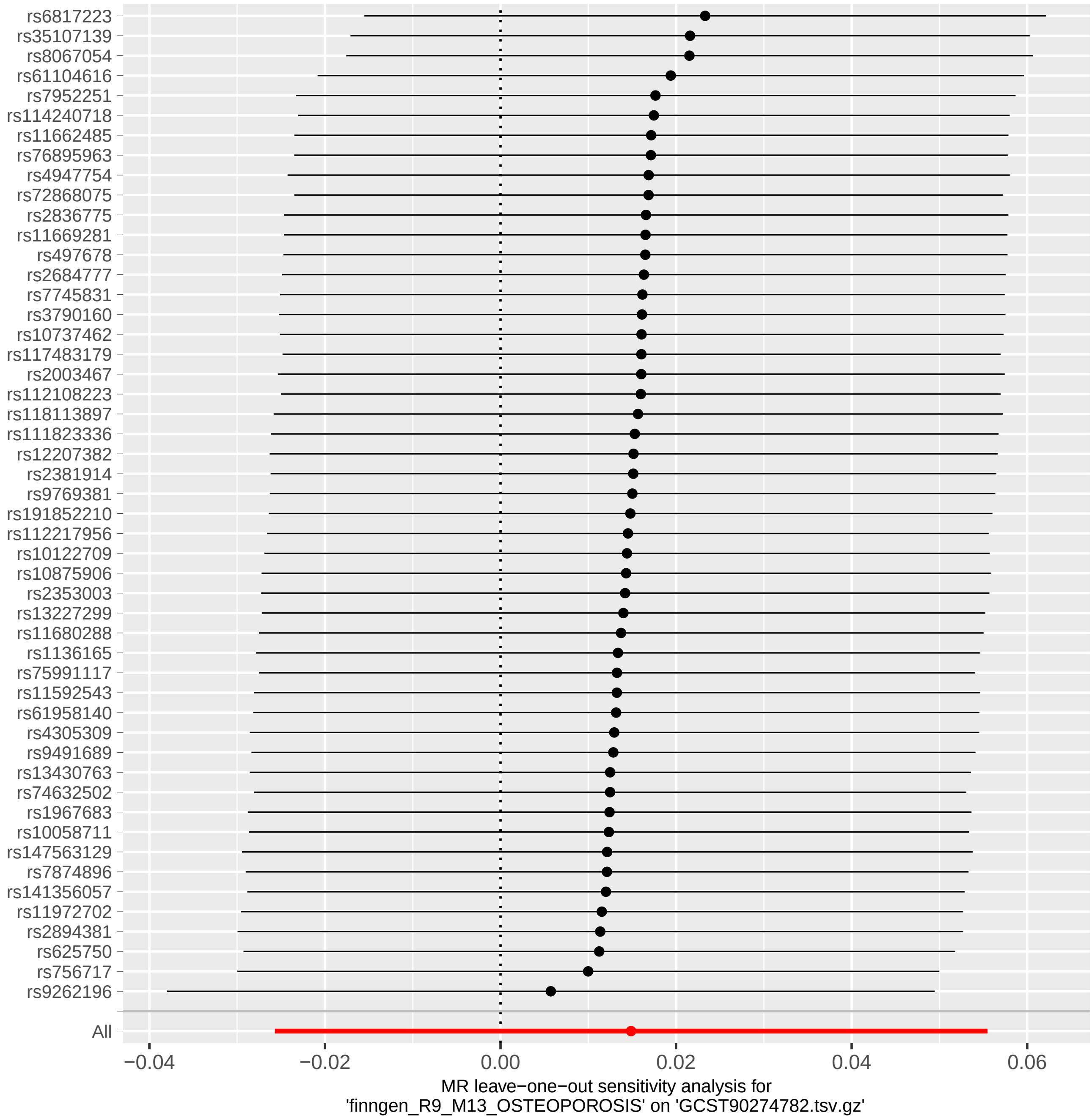
rs6817223
rs35107139
rs8067054
rs61104616
rs7952251
rs114240718
rs11662485
rs76895963
rs4947754
rs72868075
rs2836775
rs11669281
rs497678
rs2684777
rs7745831
rs3790160
rs10737462
rs117483179
rs2003467
rs112108223
rs118113897
rs111823336
rs12207382
rs2381914
rs9769381
rs191852210
rs112217956
rs10122709
rs10875906
rs2353003
rs13227299
rs11680288
rs1136165
rs75991117
rs11592543
rs61958140
rs4305309
rs9491689
rs13430763
rs74632502
rs1967683
rs10058711
rs147563129
rs7874896
rs141356057
rs11972702
rs2894381
rs625750
rs756717
rs9262196
All
−0.04
−0.02
0.00
0.02
0.04
0.06
MR leave−one−out sensitivity analysis for
'finngen_R9_M13_OSTEOPOROSIS' on 'GCST90274782.tsv.gz'
